# Prescription-Duration Patterns of Outpatient Macrolide Use in Participating Community Pharmacies in Tokyo, Japan: A 24-Month Repeated Cross-Sectional Study

**DOI:** 10.3390/idr18050120

**Published:** 2026-10-19

**Authors:** Yuki Kishino, Yoshiharu Fukuda, Shinya Kiyokawa, Kaei Hiroi

**Affiliations:** 1 PHARUMO, Inc., Tokyo 163-1452, Japan; s.kiyokawa@pharumo.jp (S.K.); k.hiroi@pharumo.jp (K.H.); 2 Graduate School of Public Health, Teikyo University, Tokyo 173-8605, Japan; fukuda.yoshiharu.li@teikyo-u.ac.jp

**Keywords:** antimicrobial stewardship, antimicrobial resistance, AWaRe, macrolide, prescription duration, community pharmacy

## Abstract

Background: Macrolides are classified in the Watch group of the World Health Organization Access, Watch, and Reserve (AWaRe) classification and are prescribed for both acute infections and chronic respiratory diseases. Aggregate antimicrobial use indicators do not distinguish short- from extended-duration prescribing. This study characterized outpatient macrolide prescription-duration patterns using community pharmacy dispensing data. Methods: A retrospective observational study comprising repeated cross-sectional analyses at the drug prescription-record level and a secondary patient-level longitudinal analysis was conducted using anonymized dispensing data from participating community pharmacies in Tokyo, Japan, collected between April 2023 and March 2025. Prescription durations were classified as 1–14, 15–28, 29–89, and ≥90 days. The primary outcome was a prescription duration of ≥29 days, corresponding to the Japanese dispensing-fee classification during the study period. Modified Poisson regression was used to estimate associations with extended-duration prescription records. Results: The analysis included 200,632 systemic macrolide drug prescription records from 139,293 pharmacy-specific patient keys. Overall, 13,555 records (6.8%) had durations of ≥29 days. The proportion increased from 1.6% among patients aged 0–14 years to 25.4% among those aged ≥75 years. Compared with patients aged 15–39 years, the adjusted prevalence ratios were 2.11 (95% CI, 1.73–2.57) for those aged 40–64 years, 4.30 (95% CI, 3.18–5.82) for those aged 65–74 years, and 5.45 (95% CI, 3.98–7.47) for those aged ≥75 years. Marked differences were observed among macrolide agents and formulations. Sensitivity analyses excluding exact duplicates, restricting the analysis to continuously contributing pharmacies, and using an alternative clustering specification supported the principal adult/older-age and agent-specific findings. Conclusions: Extended-duration macrolide dispensing records were more frequently observed in older age groups and specific erythromycin formulations among participating pharmacies. Prescription-duration and repeated-prescribing measures may complement conventional antimicrobial utilization indicators by identifying strata for subsequent indication-linked review; they should not be interpreted as stand-alone measures of prescribing appropriateness.

## 1. Introduction

Antimicrobial resistance (AMR) represents a major threat to the effective treatment of infectious diseases and constitutes an important global public health concern. The optimization of antimicrobial use is therefore required across both inpatient and outpatient healthcare settings [1,2]. Because a substantial proportion of antimicrobial agents are prescribed in ambulatory care, the systematic evaluation of outpatient prescribing patterns is essential for the development of effective antimicrobial stewardship (AMS) strategies.

The World Health Organization (WHO) Access, Watch, and Reserve (AWaRe) classification provides a standardized framework for evaluating antimicrobial use and supporting stewardship interventions [3]. Commonly prescribed systemic macrolides, including clarithromycin, azithromycin, and erythromycin, are classified within the Watch group. Antibiotics in this group have a comparatively high potential for the selection of antimicrobial resistance and should therefore be prioritized for monitoring and stewardship activities. Previous studies using national health insurance claims data have demonstrated that macrolides constitute a substantial proportion of outpatient oral antimicrobial use in Japan, together with third-generation cephalosporins and fluoroquinolones [4]. Accordingly, the characterization of macrolide prescribing patterns remains an important component of outpatient AMR surveillance in Japan.

The clinical contexts in which macrolides are prescribed are heterogeneous. These agents are administered as short-course antimicrobial therapy for acute infectious diseases, including respiratory tract infections. However, macrolides may also be prescribed for extended periods in patients with chronic respiratory disorders, including bronchiectasis and chronic inflammatory airway diseases, because of their anti-inflammatory and immunomodulatory properties [5,6,7]. Prolonged macrolide administration may provide clinical benefits in appropriately selected patients, but it may also contribute to the selection of resistant organisms and increase the risk of adverse events. Consequently, aggregate indicators such as prescription counts, defined daily doses, or days of therapy may not adequately distinguish short-course treatment for acute infections from extended-duration prescribing for chronic or non-acute conditions.

Previous Japanese studies have examined the diseases associated with macrolide prescriptions and the distribution of prescription durations using health insurance claims databases [8,9]. These studies demonstrated that the interpretation of macrolide utilization requires consideration of treatment duration. Nevertheless, evidence derived from community pharmacy dispensing data remains limited. In particular, few studies have simultaneously evaluated prescription duration according to individual macrolide agents and formulations, patient age, temporal variation, and repeated prescribing within the same patient. Such analyses may provide information that cannot be obtained from aggregate antimicrobial utilization indicators alone.

Community pharmacy dispensing data have several characteristics that support their use in outpatient antimicrobial surveillance. These data reflect medications that were dispensed to patients and can include drug codes, prescription duration, patient age categories, anonymized patient identifiers, prescribing institution identifiers, and dispensing pharmacy identifiers. Although dispensing data generally do not include clinical diagnoses, infection sites, microbiological findings, disease severity, or the prescriber’s therapeutic intent, they can be used to characterize the duration structure of outpatient antimicrobial use. Furthermore, anonymized patient identifiers permit the assessment of repeated prescriptions across multiple months within the same pharmacy, thereby complementing analyses based solely on the duration of an individual prescription.

Prescription duration may also provide a practical basis for stratifying outpatient macrolide use. During the study period, the Japanese dispensing-fee classification categorized prescriptions of 29 days or longer within the longest duration category [10]. Accordingly, a threshold of ≥29 days provides temporal consistency with the administrative classification in effect when the study data were generated. The Japanese reimbursement system was subsequently revised in fiscal year 2026, with prescriptions of ≥28 days classified as long-duration prescriptions [11]. Application of the revised ≥28-day threshold to data generated before the reform may therefore be appropriate as a sensitivity analysis rather than as the primary analytical definition. Neither threshold should be interpreted as a clinical definition of long-term macrolide therapy or as an indicator of inappropriate prescribing.

The present study used 24 months of dispensing data obtained from participating community pharmacies in Tokyo, Japan, to characterize the duration structure of outpatient systemic macrolide prescriptions. The primary objective was to determine the proportion and distribution of prescription records with durations of ≥29 days according to macrolide agent or formulation and patient age group. The secondary objectives were to evaluate monthly proportions while accounting for changes in the number of participating pharmacies, to examine repeated macrolide prescribing at the patient level, and to identify factors associated with extended-duration prescription records. A threshold of ≥28 days was evaluated in a sensitivity analysis based on the revised fiscal year 2026 dispensing-fee classification, whereas prescriptions of ≥90 days were evaluated as a secondary indicator of particularly extended duration. This study was designed to identify patient and prescription strata that may warrant subsequent indication-linked review rather than to determine the clinical appropriateness of individual prescriptions.

## 2. Materials and Methods

### 2.1. Study Design and Data Source

This retrospective observational study comprised repeated cross-sectional analyses at the drug prescription-record level and a secondary longitudinal analysis at the patient level, using anonymized dispensing data obtained from participating community pharmacies in Tokyo, Japan, between April 2023 and March 2025.

The participating pharmacies were community pharmacies connected to Pharmo’s routine data-linkage infrastructure. Anonymized dispensing data were automatically collected as part of routine pharmacy operations and were not generated specifically for this study. The pharmacies were not recruited or selected by random sampling for the present study; rather, the analytical dataset comprised data available from pharmacies contributing to the data network during the study period. Accordingly, the number and composition of participating pharmacies changed over time as network coverage expanded.

The dataset contained the dispensing month, anonymized patient identifier, age category, sex, Japanese drug code (YJ code), prescribed duration, dispensed quantity, dispensing-unit classification, anonymized prescribing institution identifier, anonymized pharmacy identifier, and prefectural code. The dataset did not contain clinical diagnoses, infection sites, laboratory findings, microbiological test results, disease severity, prescriber specialty, detailed clinical setting, or information regarding the clinical indication for each prescription.

The number of pharmacies contributing any dispensing data increased from 240 in April 2023 to 1687 in March 2025, irrespective of whether an eligible systemic macrolide record was present. Therefore, monthly prescription counts were not interpreted as direct measures of temporal changes in macrolide use. Monthly analyses were primarily based on proportions, and additional analyses were conducted among pharmacies that continuously contributed data throughout the entire 24-month study period.

### 2.2. Analytical Unit

The analytical unit was a drug prescription record. One row corresponding to one YJ code was defined as one drug prescription record.

Because the dataset did not contain a prescription-level identifier, records for multiple medications prescribed concurrently could not be aggregated into a single prescription. Accordingly, the results are presented as the number of drug prescription records rather than the number of prescription forms or clinical treatment episodes.

Only records classified as “day,” for which the prescribed duration could be evaluated in days, were included in the prescription-duration analysis. Records with other dispensing-unit classifications were excluded.

### 2.3. Identification of Systemic Macrolide Prescriptions

Systemic macrolide antibiotics were identified using the first seven digits of the YJ code. The following agents and formulations were included:

1. Clarithromycin
2. Azithromycin
3. Roxithromycin
4. Erythromycin ethylsuccinate
5. Erythromycin
6. Erythromycin stearate

The erythromycin formulations were analyzed separately because their prescription-duration distributions differed substantially. Aggregate results for all erythromycin formulations were calculated only as supplementary summaries, with the components of the combined category explicitly defined.

Topical macrolide preparations and records that could not be assigned to the prespecified systemic macrolide categories were excluded.

### 2.4. Classification of Prescription Duration

Prescription duration was categorized into four mutually exclusive groups: 1–14 days, 15–28 days, 29–89 days, and ≥90 days.

The primary outcome was a prescription-duration record of ≥29 days. This threshold was selected because prescriptions of ≥29 days corresponded to the longest dispensing-fee duration category in effect during the study period, thereby providing an administratively defined and temporally consistent cut point for stratifying prescription duration. It was not intended to represent a clinical definition of long-term macrolide therapy or a threshold of inappropriate prescribing.

A threshold of ≥28 days, corresponding to the revised Japanese dispensing-fee classification introduced in fiscal year 2026 after the study period, was evaluated as a sensitivity analysis. Prescriptions of ≥90 days were examined as a secondary indicator of particularly extended duration. This cut point corresponds to approximately three months of supply and is close to the ≥91-day cut point used in a previous Japanese claims-based study to identify long-term macrolide therapy among patients with chronic airway disease [9]; it was used here as a pragmatic descriptive marker rather than a clinical definition of long-term macrolide therapy.

### 2.5. Age Classification

Patients were classified into the following age groups:

1. 0–14 years
2. 15–39 years
3. 40–64 years
4. 65–74 years
5. ≥75 years

These categories were used to evaluate differences in prescription-duration patterns among pediatric patients, younger adults, middle-aged adults, and older populations.

Because age was recorded in predefined five-year categories, analyses were conducted using grouped rather than exact age. The consistency of anonymized patient identifiers was assessed by examining changes in sex and age category during the study period. According to the data provider, anonymized patient identifiers were designed to remain stable across dispensing months within the same pharmacy but were not linkable across different pharmacies.

For the purposes of this study, a patient was operationally defined using a pharmacy-specific patient key created by combining the anonymized pharmacy identifier and anonymized patient identifier.

### 2.6. Descriptive Analysis

The numbers and proportions of drug prescription records were calculated according to prescription-duration category, macrolide agent or formulation, age group, sex, and dispensing month.

The proportion of prescription records with a duration of ≥29 days was calculated by dividing the number of macrolide prescription records with a duration of ≥29 days by the total number of macrolide prescription records.

Agent-specific proportions were calculated using the total number of records for each macrolide agent or formulation as the denominator. Age-specific proportions were calculated using the total number of macrolide records within each age group as the denominator.

Cross-tabulations of macrolide agent or formulation and age group were prepared to determine whether age-related differences in prescription duration remained within individual drug categories.

### 2.7. Monthly Analysis

For each month, the proportions of records with durations of 1–14 days, 15–28 days, 29–89 days, and ≥90 days were calculated using the total number of macrolide drug prescription records in that month as the denominator.

Because the number of participating pharmacies changed substantially during the study period, monthly prescription counts were presented only as descriptive information regarding the size of the monthly dataset. Temporal comparisons were based primarily on prescription-duration proportions.

To evaluate the potential influence of changes in pharmacy participation, analyses were repeated among the 122 pharmacies that contributed any dispensing data in each of the 24 study months, regardless of whether an eligible macrolide record was present in every month. The distributions of age, sex, macrolide agent/formulation, and prescription duration in the fixed panel were also compared with those in the full analytical dataset (Supplementary Table S1). Because the fixed panel was a nested subset of the full dataset, these comparisons were descriptive; absolute standardized differences were used to quantify compositional differences.

### 2.8. Patient-Level Repeated-Prescribing Analysis

Anonymized patient identifiers were used to evaluate repeated macrolide prescribing during the study period. The following patient-level measures were calculated:

1. Patients with two or more macrolide drug prescription records
2. Patients with macrolide prescriptions in two or more months
3. Patients with macrolide prescriptions in three or more months
4. Patients with at least one prescription record of ≥29 days
5. Patients with at least one prescription record of ≥28 days
6. Patients with repeated short-duration prescriptions but no extended-duration prescription record

The number of distinct dispensing months was used to describe repeated prescribing. Because the dataset contained the dispensing month but not the exact dispensing date, individual treatment episodes and intervals between prescriptions could not be determined. Repeated prescribing was therefore not interpreted as continuous therapy. Repeated short-duration prescribing was defined as macrolide records observed in at least three distinct dispensing months, all with durations of 1–14 days, and with no prescription record of ≥29 days during the study period. A threshold of three distinct dispensing months was chosen as a pragmatic descriptive indicator of dispensing recurring beyond a single acute course, because a single short course would not ordinarily generate dispensing in three separate calendar months. The proportions of patients with macrolide prescriptions in at least two, six, and 12 months are also reported to show how the results vary with this cut point (Section 3.6).

### 2.9. Multivariable Analysis

Modified Poisson regression with a log link and robust variance estimation was used to estimate adjusted prevalence ratios (aPRs) and 95% confidence intervals (CIs) for factors associated with prescription records of ≥29 days.

The primary regression outcome was a binary variable indicating a prescription duration of ≥29 days. The explanatory variables were age group, macrolide agent or formulation, sex, and dispensing month. Patients aged 15–39 years were used as the reference age group, clarithromycin as the reference macrolide agent, and male patients as the reference sex category. Dispensing month was included as a categorical variable. The covariates were chosen on the basis of the demographic and prescription characteristics available in the dataset rather than by a data-driven variable selection procedure.

Two-way cluster-robust standard errors were calculated using the pharmacy-specific patient key and prescribing-institution identifier to account for correlation among repeated records from the same patient and similarities in prescribing practices within the same institution. Prescription duration is specified by the prescriber, so correlation is expected among records from the same prescribing institution; the primary model therefore clustered on the patient and the prescribing institution. Clustering by dispensing pharmacy was evaluated as an alternative specification in a sensitivity analysis (Section 2.10). The primary regression included 200,539 records, 139,241 pharmacy-specific patient clusters, and 6157 prescribing-institution clusters. No missing-data imputation was performed; records with sex values other than male or female were excluded from regression analyses.

The model was intended to describe adjusted associations within the measured dispensing variables. Because relevant clinical and prescriber-level characteristics were unavailable, the resulting estimates were not interpreted as fully confounder-adjusted causal effects.

### 2.10. Sensitivity Analyses

The robustness and interpretation of the primary findings were evaluated using the following additional analyses: (1) redefining the primary outcome as ≥28 days; (2) describing records of ≥90 days; (3) excluding exactly duplicated records and repeating the primary multivariable model (Supplementary Table S2); (4) repeating the primary multivariable model in the 122-pharmacy fixed panel (Supplementary Table S3); (5) recalculating the primary model with two-way clustering by dispensing pharmacy and prescribing institution (Supplementary Table S4); (6) comparing the composition of the full dataset and fixed panel; and (7) conducting patient-level analyses of repeated prescribing.

Exactly duplicated records were defined as rows with identical values across all available variables. There were 5577 duplicate groups comprising 6032 excess rows. Because exact dispensing dates and prescription-level identifiers were unavailable, these rows could not be definitively classified as duplicated data or separate dispensing events; they were retained in the primary analysis and excluded only in a sensitivity analysis.

Data-quality checks assessed blank values in principal analytical fields, range and format validity, within-patient sex consistency, and longitudinal consistency of the predefined age categories (Supplementary Table S5). Patient identifiers were evaluated using the pharmacy-specific composite key because identifiers were designed to be stable only within the same pharmacy.

To facilitate interpretation of effect magnitude, marginal standardized predicted prevalences were calculated from the primary modified Poisson model. For each level of age group, macrolide agent/formulation, or sex, the factor of interest was set to that level for all records while the observed values of the remaining covariates were retained. Predicted probabilities were averaged across the regression sample, and adjusted absolute prevalence differences relative to the prespecified reference category were calculated (Supplementary Table S6).

### 2.11. Statistical Analysis

Categorical variables are presented as numbers and percentages. For the chi-square analyses, the 29–89-day and ≥90-day categories were combined into a single ≥29-day category, yielding three mutually exclusive duration categories: 1–14 days, 15–28 days, and ≥29 days. Differences in this three-category distribution according to age group and macrolide agent or formulation were evaluated using the chi-square test, with Cramér’s V used to describe association magnitude.

Agent/formulation- and age-specific proportions are shown with 95% Wilson confidence intervals. Adjusted associations are presented as aPRs with 95% CIs and two-sided *p*-values. For marginal standardized predictions, 95% CIs for predicted prevalences and absolute prevalence differences were estimated using the delta method with the same two-way cluster-robust covariance matrix as the primary model. A two-sided *p*-value of <0.05 was considered statistically significant.

Primary analyses were conducted in R version 4.5.1 (R Foundation for Statistical Computing, Vienna, Austria). Additional sensitivity, quality-control, and post-estimation analyses were implemented in Python 3.13.5 (Python Software Foundation, Wilmington, DE, USA) using NumPy 2.3.5 (https://numpy.org) and SciPy 1.17.0 (https://scipy.org). These additional analyses used the same prespecified analytical definitions as the primary analysis.

### 2.12. Ethical Considerations

This study used anonymously processed secondary dispensing data and did not involve direct contact with patients or the use of personally identifiable information.

The study protocol was submitted to Teikyo University for ethical consideration. Because the dataset constituted anonymously processed information, Teikyo University determined that the study was not subject to formal review by the institutional ethics committee.

The study was conducted in accordance with the applicable institutional requirements for research using anonymously processed secondary data.

### 2.13. Reporting Guidelines

This study was reported in accordance with the Strengthening the Reporting of Observational Studies in Epidemiology (STROBE) statement and the relevant recommendations of the REporting of studies Conducted using Observational Routinely collected health Data (RECORD) statement.

## 3. Results

### 3.1. Study Population and Overall Prescription-Duration Distribution

**Figure 1:**
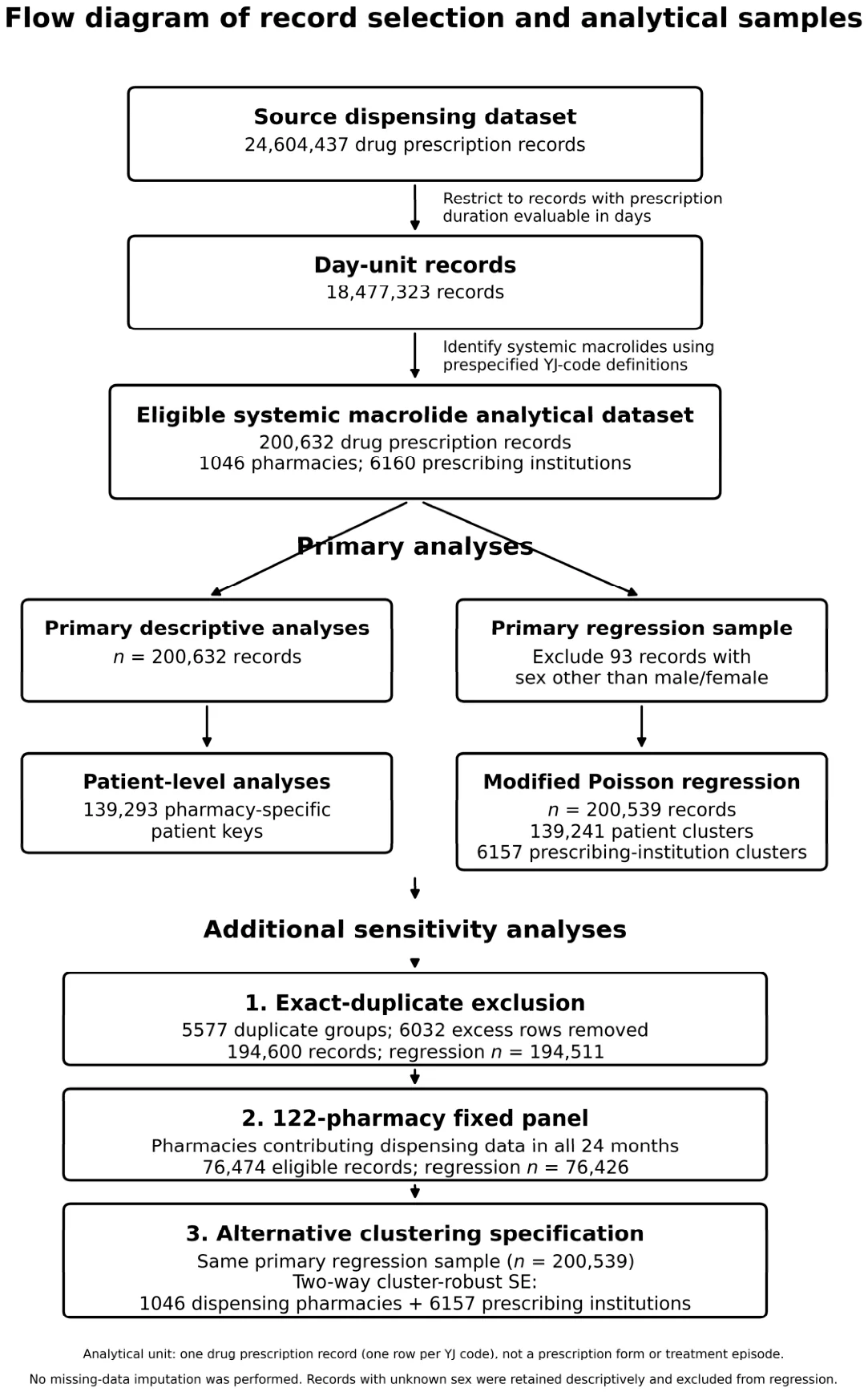
Flow diagram of record selection and analytical samples. The source dataset contained 24,604,437 drug prescription records. Records with prescription duration evaluable in days were retained, and systemic macrolides were identified using prespecified YJ-code definitions, yielding 200,632 eligible drug prescription records. The primary regression excluded 93 records with sex values other than male or female. Additional sensitivity analyses included exact-duplicate exclusion, restriction to the 122-pharmacy fixed panel, and alternative clustering.

During the 24-month study period, the source dataset contained 24,604,437 drug prescription records. Of these, 18,477,323 were classified as “day” records, for which the prescribed duration could be evaluated in days. After systemic macrolide agents were identified using the prespecified YJ code definitions, 200,632 eligible macrolide drug prescription records were included (Figure 1).

The descriptive analytical dataset comprised 139,293 pharmacy-specific patient keys. At least one eligible systemic macrolide record was identified in data from 1046 community pharmacies and 6160 prescribing institutions.

**Figure 2:**
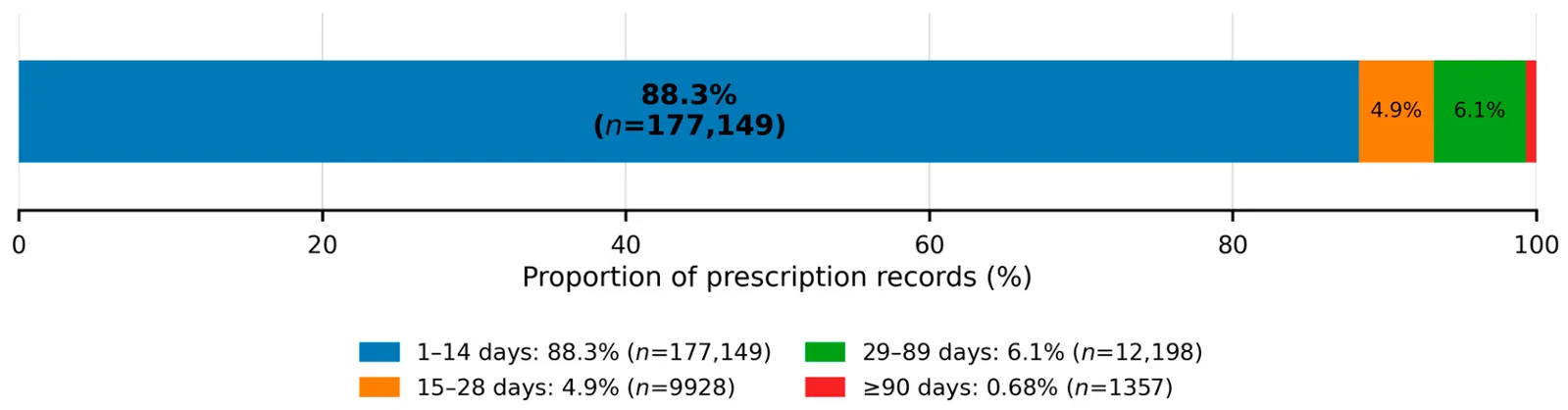
Distribution of prescription duration among systemic macrolide drug prescription records. The 100% stacked horizontal bar shows the mutually exclusive duration categories of 1–14 days, 15–28 days, 29–89 days, and ≥90 days. Percentages and record counts are shown for the 200,632 prescription records. Records with durations of ≥29 days (29–89 days and ≥90 days combined) constituted the primary outcome.

Among the 200,632 macrolide drug prescription records, 177,149 (88.3%) had durations of 1–14 days, 9928 (4.9%) had durations of 15–28 days, 12,198 (6.1%) had durations of 29–89 days, and 1357 (0.68%) had durations of ≥90 days. Overall, 13,555 records (6.8%) had durations of ≥29 days, which constituted the primary outcome (Figure 2).

### 3.2. Agent- and Formulation-Specific Prescription-Duration Patterns

Clarithromycin accounted for 144,142 records (71.8%) and was the most frequently dispensed macrolide. Of these, 9941 records had durations of ≥29 days, corresponding to a proportion of 6.9%.

Azithromycin accounted for 40,133 records (20.0%), of which 276 (0.7%) had durations of ≥29 days. Roxithromycin accounted for 10,897 records (5.4%), of which 581 (5.3%) had durations of ≥29 days.

Substantial differences were observed among erythromycin formulations. Erythromycin stearate accounted for 3610 records, of which 2322 (64.3%) had durations of ≥29 days. Erythromycin ethylsuccinate accounted for 1542 records, of which 217 (14.1%) had durations of ≥29 days. Erythromycin accounted for 308 records, of which 218 (70.8%) had durations of ≥29 days.

**Figure 3:**
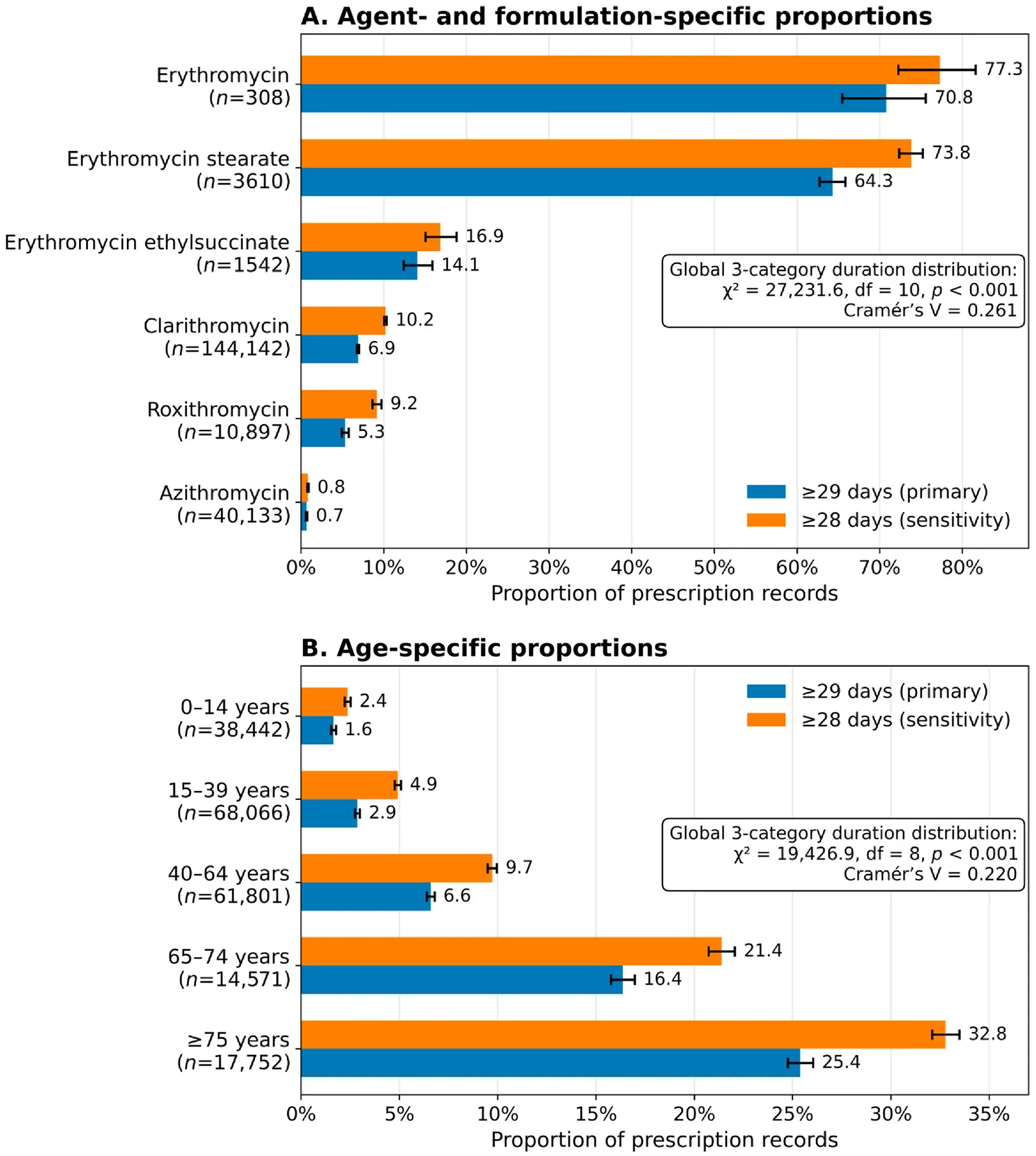
Proportions of extended-duration prescriptions according to macrolide agent or formulation and patient age group. (**A**) Proportions of prescription records with durations of ≥29 days (primary outcome) and ≥28 days (sensitivity-analysis outcome) according to macrolide agent or formulation. Categories are ordered by the proportion with durations of ≥29 days. (**B**) Corresponding proportions according to patient age group. Error bars indicate 95% Wilson confidence intervals for the binomial proportions. Statistical annotations report the global chi-square test and Cramér’s V for the mutually exclusive three-category duration distribution (1–14 days, 15–28 days, and ≥29 days), rather than a direct comparison between the ≥29-day and ≥28-day thresholds. Records with durations of ≥29 days are included within the ≥28-day category. The duration thresholds are administrative and analytical classifications and should not be interpreted as clinical definitions of long-term macrolide therapy.

When the three erythromycin formulations were combined, they accounted for 5460 records, including 2757 records (50.5%) with durations of ≥29 days (Table 1, Panel A; Figure 3A). The overall three-category prescription-duration distribution differed significantly according to macrolide agent or formulation (χ^2^ = 27,231.6, degrees of freedom = 10, *p* < 0.001; Cramér’s V = 0.261).

### 3.3. Age-Specific Prescription-Duration Patterns

The proportion of records with durations of ≥29 days increased across age groups. Among patients aged 0–14 years, 633 of 38,442 records (1.6%) had durations of ≥29 days. The corresponding proportions were 1950 of 68,066 records (2.9%) among patients aged 15–39 years, 4078 of 61,801 records (6.6%) among those aged 40–64 years, 2385 of 14,571 records (16.4%) among those aged 65–74 years, and 4509 of 17,752 records (25.4%) among those aged ≥75 years.

The three-category prescription-duration distribution differed significantly according to age group (χ^2^ = 19,426.9, degrees of freedom = 8, *p* < 0.001; Cramér’s V = 0.220).

Using the sensitivity-analysis threshold of ≥28 days, the corresponding proportions were 2.4%, 4.9%, 9.7%, 21.4%, and 32.8% across the five age groups, respectively (Table 1, Panel B; Figure 3B). An age-related gradient was also observed when the analysis was restricted to clarithromycin, with the proportion of records with durations of ≥29 days increasing from 1.9% among patients aged 0–14 years to 21.9% among those aged ≥75 years.

### 3.4. Factors Associated with Prescription Durations of ≥29 Days

The modified Poisson regression analysis included 200,539 records after excluding 93 records in which sex was not recorded as male or female. The model included age group, macrolide agent or formulation, sex, and dispensing month as explanatory variables. Two-way cluster-robust standard errors were calculated using the pharmacy-specific patient key and prescribing institution identifier.

Compared with patients aged 15–39 years, patients aged 0–14 years had a lower adjusted prevalence of prescription records with durations of ≥29 days (adjusted prevalence ratio [aPR], 0.50; 95% confidence interval [CI], 0.33–0.75; *p* < 0.001). The adjusted prevalence was higher among patients aged 40–64 years (aPR, 2.11; 95% CI, 1.73–2.57; *p* < 0.001), those aged 65–74 years (aPR, 4.30; 95% CI, 3.18–5.82; *p* < 0.001), and those aged ≥75 years (aPR, 5.45; 95% CI, 3.98–7.47; *p* < 0.001).

Using clarithromycin as the reference category, azithromycin was associated with a lower adjusted prevalence of prescription records with durations of ≥29 days (aPR, 0.11; 95% CI, 0.05–0.22; *p* < 0.001). No statistically significant difference was observed between roxithromycin and clarithromycin (aPR, 0.97; 95% CI, 0.62–1.52; *p* = 0.899).

Compared with clarithromycin, erythromycin ethylsuccinate (aPR, 2.70; 95% CI, 1.63–4.45; *p* < 0.001), erythromycin (aPR, 4.57; 95% CI, 3.58–5.83; *p* < 0.001), and erythromycin stearate (aPR, 4.05; 95% CI, 3.26–5.05; *p* < 0.001) were associated with higher adjusted prevalences of prescription records with durations of ≥29 days.

Female patients had a lower adjusted prevalence of prescription records with durations of ≥29 days than male patients (aPR, 0.84; 95% CI, 0.76–0.92; *p* < 0.001) (Table 2).

Marginal standardization showed that the adjusted predicted prevalence of ≥29-day records was 3.26% among patients aged 15–39 years, 6.86% among those aged 40–64 years, 14.02% among those aged 65–74 years, and 17.76% among those aged ≥75 years. Relative to the 15–39-year reference group, the adjusted absolute prevalence differences were +3.60 percentage points (95% CI, +2.64 to +4.57), +10.76 points (+8.06 to +13.45), and +14.50 points (+11.67 to +17.33), respectively. The adjusted absolute difference for female versus male patients was −1.20 percentage points (95% CI, −1.85 to −0.56), illustrating that statistically significant associations varied substantially in absolute magnitude (Supplementary Table S6).

### 3.5. Monthly Prescription-Duration Proportions

The number of pharmacies contributing data increased from 240 in April 2023 to 1687 in March 2025. Accordingly, the number of monthly macrolide records increased with the expansion of data coverage, and monthly prescription counts were not used as the principal measure of temporal change.

Among all participating pharmacies, the monthly proportion of macrolide records with durations of ≥29 days ranged from 4.6% to 9.6%. Among the 122 pharmacies that continuously contributed data throughout all 24 months, the monthly proportion ranged from 4.9% to 10.1%.

**Figure 4:**
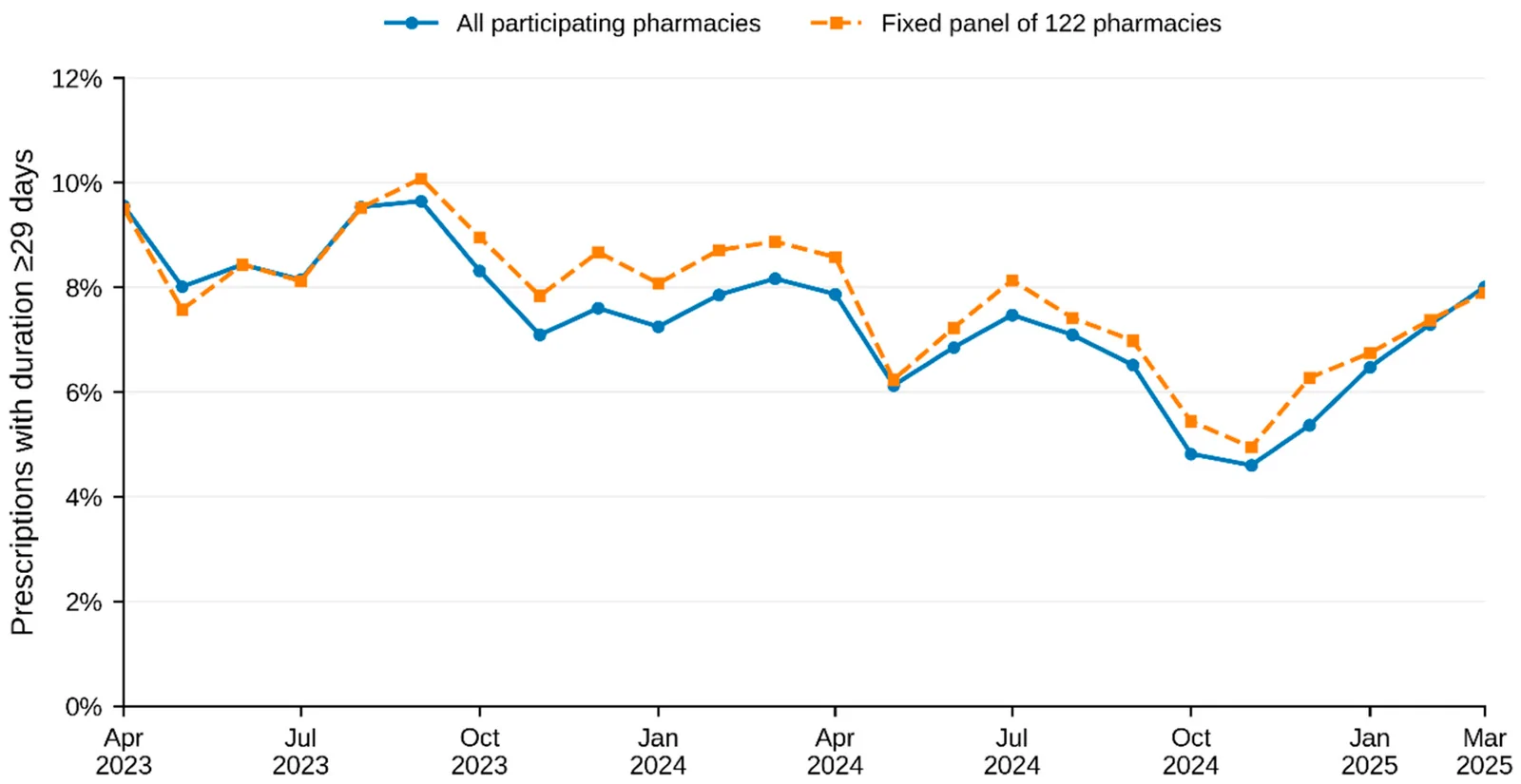
Monthly proportions of systemic macrolide prescription records with durations of ≥29 days. Results are shown for all participating community pharmacies and for the fixed panel of 122 pharmacies that contributed data throughout the complete 24-month study period. The monthly denominator was the total number of macrolide prescription records observed in the corresponding pharmacy group.

The monthly proportions obtained from the continuously participating pharmacy panel were generally similar to those obtained from all participating pharmacies, although differences between the two series were observed in individual months (Figure 4).

### 3.6. Patient-Level Repeated Prescribing

Among the 139,293 patients included in the study, 29,896 (21.5%) had two or more macrolide drug prescription records. A total of 24,690 patients (17.7%) received macrolide prescriptions in at least two distinct months, 8876 (6.4%) in at least three months, 1610 (1.2%) in at least six months, and 306 (0.2%) in at least 12 months.

At least one prescription record with a duration of ≥29 days was identified in 5468 patients (3.9%). In comparison, 5492 patients (3.9%) had macrolide records in at least three distinct months while all of their individual prescription records had durations of 1–14 days. Among these patients, 477 had short-duration records in at least six distinct months.

Because exact dispensing dates were unavailable, repeated records across different months could not be classified as continuous treatment, recurrent treatment for the same condition, or separate treatment episodes. Because patient identifiers could not be linked across pharmacies, patients who used more than one pharmacy would be counted under separate pharmacy-specific keys, and the proportions of patients with repeated or extended-duration prescribing may therefore be underestimated.

### 3.7. Sensitivity and Secondary Analyses

When the threshold was changed from ≥29 days to ≥28 days, 19,207 records (9.6%) met the outcome definition; 5652 records (2.8% of all macrolide records) had a duration of exactly 28 days. The direction of the principal adjusted associations with age group and macrolide agent/formulation remained consistent. A total of 1357 records (0.68%) had durations of ≥90 days.

Exactly duplicated records comprised 5577 duplicate groups and 6032 excess rows. After exclusion of the excess rows, 194,600 records remained; 194,511 records were included in the regression after sex-category exclusions. The adult/older-age, agent/formulation, and sex estimates remained similar to the primary model: all adjusted prevalence ratios shown in Table 2 changed by less than 7% in relative terms (for example, ≥75 vs. 15–39 years, 5.81 [95% CI, 4.24–7.96] vs. 5.45 [95% CI, 3.98–7.47]; erythromycin vs. clarithromycin, 4.27 [95% CI, 3.36–5.44] vs. 4.57 [95% CI, 3.58–5.83]), with no reversal of direction or change in statistical significance (Supplementary Table S2).

The 122-pharmacy fixed panel contained 76,474 eligible records from 47,377 pharmacy-specific patient keys and 2586 prescribing institutions; 76,426 records, 47,349 patient clusters, and 2582 prescribing-institution clusters were included in the fixed-panel regression. The principal adult/older-age and agent/formulation-specific associations remained evident, although the estimate for patients aged 0–14 years was attenuated (aPR, 0.85; 95% CI, 0.50–1.46) and was no longer statistically significant (Supplementary Table S3).

Using dispensing pharmacy and prescribing institution rather than patient and prescribing institution as the two clustering dimensions did not materially change the point estimates or statistical-significance pattern (Supplementary Table S4).

The fixed panel retained 38.1% of eligible macrolide records and was broadly similar but not identical in composition to the full dataset. It included a somewhat higher proportion of records from patients aged ≥75 years (10.9% vs. 8.8%), more azithromycin records (23.6% vs. 20.0%), fewer clarithromycin records (68.6% vs. 71.8%), and a modestly higher proportion of ≥29-day records (7.5% vs. 6.8%). The largest absolute standardized difference among the displayed record-level characteristics was 0.088 (Supplementary Table S1). Table 3 summarizes this comparison together with the adjusted prevalence ratios in the fixed panel. The age-related gradient among patients aged ≥40 years was preserved in the fixed panel, with similar or larger point estimates and wider confidence intervals, and the agent/formulation-specific estimates were directionally consistent (Table 3; Supplementary Table S3).

No principal analytical field was blank. Sex was coded as unknown in 93 records (0.046%); these records were retained descriptively and excluded from regression analyses without imputation. Longitudinal checks found no male-to-female or female-to-male switches within pharmacy-specific patient keys. Age-category changes were limited to adjacent five-year categories, with no decreases over time and no patient age range exceeding five years. Additional quality-control findings are provided in Supplementary Table S5.

## 4. Discussion

This study characterized the prescription-duration structure of outpatient systemic macrolide dispensing using 24 months of data from participating community pharmacies in Tokyo. Most drug prescription records were short-duration, whereas a smaller but clinically heterogeneous subset had durations of ≥29 days. Extended-duration records were concentrated in older age groups and specific erythromycin formulations, while clarithromycin contributed the largest absolute number of ≥29-day records because of its much greater overall use. Patient-level analyses further showed that repeated macrolide exposure may arise not only from individually extended prescriptions but also from multiple short-duration prescriptions across different months. Together, these findings suggest that prescription duration and repeated dispensing can provide information that is not captured by aggregate utilization indicators alone.

The ≥29-day threshold was selected because it corresponded to the longest dispensing-fee duration category in effect during the study period [10]. It therefore served as an administratively defined and temporally consistent cut point rather than a clinical definition of long-term macrolide therapy. The ≥28-day sensitivity analysis increased the proportion of qualifying records from 6.8% to 9.6%, largely because 5652 records were exactly 28 days, but the direction of the principal adjusted associations remained similar. Marked heterogeneity was also observed across agents and formulations. Azithromycin was predominantly dispensed for short durations, whereas several erythromycin formulations had much higher proportions of extended-duration records. At the same time, clarithromycin accounted for the largest absolute number of such records. These findings indicate that both proportional concentration and absolute prescribing volume should be considered when selecting targets for subsequent stewardship review.

A clear age-related pattern was observed. After adjustment for macrolide agent/formulation, sex, and dispensing month, the adjusted prevalence of ≥29-day records was higher in older age groups than in the 15–39-year reference group. Post-estimation standardization further showed that the adjusted predicted prevalence increased from approximately 3.3% in the 15–39-year reference group to 17.8% among patients aged ≥75 years, an adjusted absolute difference of approximately 14.5 percentage points. This pattern is consistent with previous Japanese claims-based findings showing greater contributions of extended-duration macrolide therapy in older populations [9]. International data also suggest that prolonged antibiotic exposure occurs in selected chronic respiratory populations, such as patients with chronic obstructive pulmonary disease; however, direct numerical comparisons are inappropriate because published studies have used diagnosis-specific cohorts and substantially longer definitions of long-term treatment [12]. Importantly, the observed association should not be interpreted as an independent causal effect of age. Older age may instead mark unmeasured differences in chronic respiratory disease, multimorbidity, treatment indication, or patterns of outpatient follow-up.

The patient-level analysis complements the record-level duration analysis. Some patients had at least one ≥29-day record, whereas others received only 1–14-day prescriptions but had macrolide records in three or more distinct months. Without exact dispensing dates and clinical indications, these patterns cannot be distinguished as recurrent acute treatment, intermittent prophylaxis, separate treatment episodes, or fragmented continuous therapy. Nevertheless, they demonstrate that the duration of a single prescription may not fully characterize repeated antimicrobial exposure. This distinction is also relevant to AWaRe-based assessment because clinically indicated long-term macrolide therapy, involving agents in the Watch group, can influence aggregate Access proportions [3,9]. Duration-stratified and patient-level repetition measures may therefore complement rather than replace conventional AWaRe, prescription-count, DDD, or DOT indicators.

The substantial increase in participating pharmacies during the study period required explicit consideration of changing data coverage. The participating pharmacies were not selected through probability sampling but were pharmacies connected to Pharmo’s routine data-linkage infrastructure, and both their number and composition changed as network coverage expanded. Restriction to the 122 pharmacies contributing data in all 24 months retained the principal adult/older-age and agent-specific associations, although the pediatric estimate was attenuated. The fixed panel was broadly similar but not identical to the full analytical dataset and therefore does not eliminate selection bias. Accordingly, the findings should be interpreted as dispensing-duration patterns observed among participating community pharmacies rather than as population-representative estimates for all pharmacies or residents of Tokyo.

These findings have practical implications for community-based antimicrobial stewardship. Prescription-duration distributions could be used by community pharmacists and regional AMS teams to identify drug, age, or repeated-prescribing strata with either a high proportion or a high absolute number of extended-duration records. Such strata could then be prioritized for indication-linked review, including confirmation of treatment purpose, assessment of continued necessity, and verification of appropriate monitoring when prolonged therapy is intended. Pharmacist-prescriber communication may be particularly useful when extended-duration or repeated macrolide dispensing recurs over multiple months. International stewardship research has also emphasized prescription duration as an important outpatient quality dimension [13]. However, the present data should be used as a screening and prioritization tool rather than as a stand-alone measure of prescribing appropriateness, because international respiratory guidance recognizes that long-term macrolides can be appropriate for selected patients, including those with bronchiectasis at high risk of exacerbations [5,14].

This study has several strengths. It included more than 200,000 macrolide drug prescription records from more than 139,000 pharmacy-specific patient keys over 24 months and incorporated prescription duration, individual agents and formulations, age group, dispensing month, prescribing-institution clustering, and patient-level repetition. Robustness was examined using alternative duration thresholds, exclusion of exactly duplicated records, a continuously contributing pharmacy panel, and an alternative clustering specification. In addition, absolute standardized prevalence differences were calculated to distinguish practically large associations from statistically significant but smaller effects.

Several limitations should be considered. First, the dispensing data did not contain diagnoses, prescribing indications, prescriber specialty, detailed clinical setting, infection site, disease severity, microbiological findings, comorbidities, treatment response, adherence, or clinical outcomes. Consequently, the study could not determine whether an extended-duration record represented clinically appropriate maintenance therapy or potentially unnecessary prolonged treatment. Differences in prescription duration across age groups, agents, or formulations therefore cannot be interpreted as evidence of inappropriate prescribing. Residual confounding is therefore likely, and the adjusted prevalence ratios should be interpreted as associations within the measured dispensing variables rather than causal effects. Future linkage with diagnostic, electronic health record, or other clinical data will be required for indication-specific evaluation. Second, the ≥29-day threshold was administrative rather than clinical, and its prevalence should not be compared directly with studies using thresholds such as >14 days, ≥91 days, or >6 months [8,9,12].

Additional data-structure limitations also apply. Exact dispensing dates were unavailable, preventing construction of continuous treatment episodes and assessment of intervals between prescriptions. The absence of a prescription-level identifier meant that records for multiple medications on the same prescription could not be combined, and exactly matching records could not be definitively classified as duplicated data or separate dispensing events. Patient identifiers were stable only within the same pharmacy, so dispensing at other pharmacies could not be attributed to the same individual and repeated exposure may have been underestimated. Finally, because participating pharmacies were not randomly selected and changed over time, the results cannot be assumed to represent all community pharmacies in Tokyo, even though the fixed-panel and other sensitivity analyses supported the principal findings.

In summary, duration-stratified community-pharmacy data identified distinct patterns of outpatient macrolide dispensing by age group, agent/formulation, and repeated prescribing. These patterns may complement conventional antimicrobial utilization metrics by identifying strata for subsequent indication-linked clinical review. Their interpretation should remain descriptive and non-causal until dispensing information can be linked with diagnostic, clinical, microbiological, and outcome data.

## 5. Conclusions

This study characterized dispensing-duration patterns of outpatient systemic macrolide use using 24 months of data from participating community pharmacies in Tokyo, Japan. Most drug prescription records were short-duration, whereas ≥29-day records were more frequently observed in older age groups and specific erythromycin formulations. These associations persisted after adjustment for the measured dispensing variables but should not be interpreted causally or as evidence of inappropriate prescribing, because diagnosis, clinical indication, comorbidities, and prescriber specialty were unavailable.

Prescription-duration and repeated-prescribing measures may complement conventional antimicrobial utilization indicators by identifying strata for subsequent indication-linked review. Integration with diagnostic, clinical, microbiological, and outcome data is required to assess the appropriateness and consequences of extended-duration macrolide use.

## Figures and Tables

**Table 1:** Extended-duration systemic macrolide prescription records according to agent or formulation and patient age group. Panel (**A**). Macrolide agent or formulation. Panel (**B**). Patient age group.

**(A)**			
**Agent or Formulation**	**Total Records, *n***	**≥29 Days, *n* (%)**	**≥28 Days, *n* (%)**
Clarithromycin	144,142	9941 (6.9)	14,712 (10.2)
Azithromycin	40,133	276 (0.7)	333 (0.8)
Roxithromycin	10,897	581 (5.3)	999 (9.2)
Erythromycin stearate	3610	2322 (64.3)	2665 (73.8)
Erythromycin ethylsuccinate	1542	217 (14.1)	260 (16.9)
Erythromycin	308	218 (70.8)	238 (77.3)
**(B)**			
**Age Group**	**Total Records, *n***	**≥29 Days, *n* (%)**	**≥28 Days, *n* (%)**
0–14 years	38,442	633 (1.6)	909 (2.4)
15–39 years	68,066	1950 (2.9)	3350 (4.9)
40–64 years	61,801	4078 (6.6)	6010 (9.7)
65–74 years	14,571	2385 (16.4)	3117 (21.4)
≥75 years	17,752	4509 (25.4)	5821 (32.8)

Note: The ≥29-day threshold was the primary outcome, and the ≥28-day threshold was used for sensitivity analysis. Records with durations of ≥29 days are included within the ≥28-day counts. Percentages are calculated using the total number of records in each row.

**Table 2:** Factors associated with systemic macrolide prescription records with durations of ≥29 days.

Variable	Category	aPR	95% CI	*p*-Value
Age group	15–39 years	Reference	—	—
	0–14 years	0.50	0.33–0.75	<0.001
	40–64 years	2.11	1.73–2.57	<0.001
	65–74 years	4.30	3.18–5.82	<0.001
	≥75 years	5.45	3.98–7.47	<0.001
Agent or formulation	Clarithromycin	Reference	—	—
	Azithromycin	0.11	0.05–0.22	<0.001
	Roxithromycin	0.97	0.62–1.52	0.899
	Erythromycin ethylsuccinate	2.70	1.63–4.45	<0.001
	Erythromycin	4.57	3.58–5.83	<0.001
	Erythromycin stearate	4.05	3.26–5.05	<0.001
Sex	Male	Reference	—	—
	Female	0.84	0.76–0.92	<0.001

Abbreviations: aPR, adjusted prevalence ratio; CI, confidence interval. Modified Poisson regression included age group, macrolide agent or formulation, sex, and dispensing month. Dispensing month was included as a categorical variable. Records with sex values other than male or female were excluded. Two-way cluster-robust standard errors were calculated using 139,241 pharmacy-specific patient clusters and 6157 prescribing-institution clusters.

**Table 3:** Comparison of the full analytical dataset and the 122-pharmacy fixed panel, with adjusted prevalence ratios for ≥29-day records.

Characteristic	Full Analytical Dataset	122-Pharmacy Fixed Panel
Eligible records (descriptive dataset), *n*	200,632	76,474
Pharmacy-specific patient keys (descriptive dataset), *n*	139,293	47,377
Prescribing institutions (descriptive dataset), *n*	6160	2586
Records from patients aged ≥75 years, %	8.8	10.9
Clarithromycin records, %	71.8	68.6
Azithromycin records, %	20.0	23.6
Records with durations of ≥29 days, %	6.8	7.5
Records in the regression sample, *n*	200,539	76,426
aPR (95% CI): 0–14 vs. 15–39 years	0.50 (0.33–0.75)	0.85 (0.50–1.46)
aPR (95% CI): 40–64 vs. 15–39 years	2.11 (1.73–2.57)	2.65 (2.01–3.48)
aPR (95% CI): 65–74 vs. 15–39 years	4.30 (3.18–5.82)	5.92 (4.12–8.52)
aPR (95% CI): ≥75 vs. 15–39 years	5.45 (3.98–7.47)	6.60 (4.22–10.34)

Abbreviations: aPR, adjusted prevalence ratio; CI, confidence interval. Descriptive counts are shown before, and regression counts after, exclusion of records with sex values other than male or female. Adjusted prevalence ratios were estimated with the primary modified Poisson model specification (Table 2) in each sample and are shown relative to the 15–39-year reference group. Full estimates are provided in Supplementary Tables S1 and S3.

## Data Availability

The data analyzed in this study contain non-public dispensing information provided under a data-use agreement and are not publicly available. Aggregated data supporting the findings of this study may be made available by the corresponding author upon reasonable request, subject to approval by the data provider and applicable data-governance requirements.

## Supplementary Materials

The following supporting information can be downloaded at https://www.mdpi.com/article/10.3390/idr18050120/s1: Table S1: Comparison of the full analytical dataset and the 122-pharmacy fixed panel; Table S2: Modified Poisson regression after exclusion of exactly duplicated records (*n* = 194,511); Table S3: Modified Poisson regression in the 122-pharmacy fixed panel (*n* = 76,426); Table S4: Primary model with alternative two-way clustering by dispensing pharmacy and prescribing institution; Table S5: Data-quality and consistency checks; Table S6: Marginal standardized predicted prevalence of ≥29-day records.

## Abbreviations

AMR	antimicrobial resistance
AMS	antimicrobial stewardship
aPR	adjusted prevalence ratio
AWaRe	Access, Watch, and Reserve
CI	confidence interval
RECORD	REporting of studies Conducted using Observational Routinely collected health Data
STROBE	Strengthening the Reporting of Observational Studies in Epidemiology
WHO	World Health Organization
YJ code	Japanese pharmaceutical product code
