# Supplementary material for: Prescription-Duration Patterns of Outpatient Macrolide Use in Participating Community Pharmacies in Tokyo, Japan: A 24-Month Repeated Cross-Sectional Study
Source: Infect Dis Rep. 2026 Oct 19;18(5):120. doi: 10.3390/idr18050120 (PMC13704055; doi:10.3390/idr18050120)
Supplement: Supplementary file 1 [file idr-18-00120-s001.zip › idr-4502319-supplementary.pdf]

Supplementary Material
Prescription-Duration Patterns of Outpatient Macrolide Use in Participating Community Pharmacies in Tokyo, Japan

Supplementary Table S1. Comparison of the full analytical dataset and the 122-pharmacy fixed panel

Characteristic	Full dataset	122-pharmacy fixed panel	Comment
Eligible records (descriptive)	200,632	76,474	Fixed panel retained 38.1% of eligible records
Pharmacy-specific patient keys	139,293	47,377	Before sex exclusion
Prescribing institutions	6160	2586	Before sex exclusion
Regression records	200,539	76,426	After sex exclusion
Patient clusters (regression)	139,241	47,349	Regression sample
Prescribing-institution clusters	6157	2582	Regression sample
Age ≥75 years	8.8%	10.9%	Fixed panel higher
Clarithromycin	71.8%	68.6%	Fixed panel lower
Azithromycin	20.0%	23.6%	Fixed panel higher
≥29-day records	6.8%	7.5%	Fixed panel modestly higher
Maximum absolute standardized difference	—	0.088	Azithromycin; all displayed values <0.10

Supplementary Table S2. Modified Poisson regression after exclusion of exactly duplicated records (n=194,511)

Comparison	aPR	95% CI
0–14 vs 15–39	0.498	0.331–0.749
40–64 vs 15–39	2.155	1.762–2.635
65–74 vs 15–39	4.539	3.355–6.141
≥75 vs 15–39	5.810	4.240–7.961
Azithromycin vs clarithromycin	0.107	0.053–0.216
Roxithromycin vs clarithromycin	0.972	0.620–1.524
Erythromycin ethylsuccinate vs clarithromycin	2.764	1.721–4.440
Erythromycin vs clarithromycin	4.274	3.359–5.438
Erythromycin stearate vs clarithromycin	3.955	3.198–4.891
Female vs male	0.840	0.764–0.923

Supplementary Table S3. Modified Poisson regression in the 122-pharmacy fixed panel (n=76,426)

Comparison	aPR	95% CI
0–14 vs 15–39	0.852	0.497–1.459
40–64 vs 15–39	2.648	2.014–3.483
65–74 vs 15–39	5.921	4.116–8.517
≥75 vs 15–39	6.604	4.220–10.336
Azithromycin vs clarithromycin	0.094	0.042–0.215
Roxithromycin vs clarithromycin	1.114	0.484–2.567
Erythromycin ethylsuccinate vs clarithromycin	3.612	2.039–6.400
Erythromycin vs clarithromycin	4.715	3.129–7.105
Erythromycin stearate vs clarithromycin	4.172	2.914–5.973
Female vs male	0.845	0.735–0.971

Supplementary Table S4. Primary model with alternative two-way clustering by dispensing pharmacy and prescribing institution

Comparison	aPR	95% CI
0–14 vs 15–39	0.497	0.337–0.733
40–64 vs 15–39	2.106	1.726–2.569
65–74 vs 15–39	4.303	3.213–5.764
≥75 vs 15–39	5.453	3.964–7.502
Azithromycin vs clarithromycin	0.111	0.055–0.224
Roxithromycin vs clarithromycin	0.971	0.626–1.507
Erythromycin ethylsuccinate vs clarithromycin	2.698	1.616–4.502
Erythromycin vs clarithromycin	4.567	3.544–5.886
Erythromycin stearate vs clarithromycin	4.055	3.228–5.093
Female vs male	0.839	0.768–0.918

Supplementary Table S5. Data-quality and consistency checks

Check	Result	Interpretation
Blank values in principal analytical fields	0	No blank values among month, patient ID, age, sex, YJ code, days, quantity, unit, prescriber ID, pharmacy ID, prefectural code
Sex coded as unknown	93 records (0.046%)	Retained descriptively; excluded from regression; no imputation
Pharmacy-specific patient keys	139,293	52 patients had unknown sex only, explaining 139,293 descriptive vs 139,241 regression patient clusters
Age validity	0–90 years	All records within prespecified range and five-year categories
Days validity	1–198	All positive integer values
Within-patient sex switches	0	No male↔female switches
Patients spanning >5 years of age categories	0	No age decreases over time
Adjacent age-category change	2,289 patients (1.64%)	Compatible with aging across five-year categories
Same-month adjacent age categories	76 patients (0.055%)	Exact dates unavailable; retained

Supplementary Table S6. Marginal standardized predicted prevalence of ≥29-day records

Category	Adjusted predicted prevalence (95% CI)	Absolute difference vs reference	95% CI of difference
Age 0–14	1.62% (1.1–2.4%)	−1.64 pp	−2.6 to −0.6 pp
Age 15–39	3.26% (2.4–4.5%)	Reference	—
Age 40–64	6.86% (5.5–8.6%)	+3.60 pp	2.6 to 4.6 pp
Age 65–74	14.02% (11.4–17.3%)	+10.76 pp	8.1 to 13.5 pp
Age ≥75	17.76% (15.0–21.0%)	+14.50 pp	11.7 to 17.3 pp
Clarithromycin	6.85% (5.5–8.6%)	Reference	—
Azithromycin	0.76% (0.4–1.5%)	−6.09 pp	−7.7 to −4.5 pp
Roxithromycin	6.65% (4.5–9.9%)	−0.20 pp	−3.2 to 2.8 pp
Erythromycin ethylsuccinate	18.47% (11.3–30.1%)	+11.62 pp	2.7 to 20.5 pp
Erythromycin	31.27% (26.0–37.6%)	+24.43 pp	18.9 to 29.9 pp
Erythromycin stearate	27.77% (24.2–31.8%)	+20.92 pp	17.3 to 24.5 pp
Male	7.49% (6.3–8.8%)	Reference	—
Female	6.29% (5.2–7.6%)	−1.20 pp	−1.8 to −0.6 pp

Note: Adjusted predictions were standardized over the observed covariate distribution of the primary regression sample. Confidence intervals were calculated using the delta method with the primary two-way cluster-robust covariance matrix.
